# Bioinformatic analysis of PD-1 checkpoint blockade response in influenza infection

**DOI:** 10.1186/s12863-022-01081-7

**Published:** 2022-08-13

**Authors:** Huilin Ou, Keda Chen, Linfang Chen, Hongcheng Wu

**Affiliations:** 1 Ningbo Medical Centre, Li Huili Hospital affiliated of Ningbo University, Ningbo, 315040 Zhejiang China; 2 Shulan International Medical College, Zhejiang Shuren University, Hangzhou, 310015 China; 3 State Key Laboratory for Diagnosis and Treatment of Infectious Diseases, First Affiliated Hospital, Zhejiang University School of Medicine, Hangzhou, 310015 China

**Keywords:** PD-1/PD-L1, Influenza, Transcriptome

## Abstract

**Background:**

The programmed cell death 1 (PD-1)/PD-1 ligand 1 (PD-L1) signaling pathway is significantly upregulated in influenza virus infection, which impairs the antiviral response. Blocking this signaling pathway may reduce the damage, lower the virus titer in lung tissue, and alleviate the symptoms of infection to promote recovery. In addition to the enhanced viral immune response, using of immune checkpoint inhibitors in influenza virus infection is controversial, the aim of this study was to identify the key factors and regulatory mechanisms in the PD-1 checkpoint blockade response microenvironment in influenza infection.

**Methods:**

A BALB/c mouse model of influenza A/PR8(H1N1) infection was established then constructed, and whole-transcriptome sequencing including mRNAs, miRNAs (microRNAs), lncRNAs (long noncoding RNAs), and circRNAs (circular RNAs) of mice treated with PD-1 checkpoint blockade by antibody treatment and IgG2a isotype control before infection with A/PR8(H1N1) were performed. Subsequently, the differential expression of transcripts between these two groups was analyzed, followed by functional interaction prediction analysis to investigate gene-regulatory circuits.

**Results:**

In total, 84 differentially expressed dif-mRNAs, 36 dif-miRNAs, 90 dif-lncRNAs and 22 dif-circRNAs were found in PD-1 antagonist treated A/PR8(H1N1) influenza-infected lungs compared with the controls (IgG2a isotype control treated before infection). In spleens between the above two groups, 45 dif-mRNAs, 36 dif-miRNAs, 57 dif-lncRNAs, and 24 dif-circRNAs were identified. Direct function enrichment analysis of dif-mRNAs and dif-miRNAs showed that these genes were mainly involved in myocardial damage related to viral infection, mitogen activated protein kinase (MAPK) signaling pathways, RAP1 (Ras-related protein 1) signaling pathway, and Axon guidance. Finally, 595 interaction pairs were obtained for the lungs and 462 interaction pairs for the spleens were obtained in the competing endogenous RNA (ceRNA) complex network, in which the downregulated mmu-miR-7043-3p and Vps39–204 were enriched significantly in PD-1 checkpoint blockade treated A/PR8(H1N1) infection group.

**Conclusions:**

The present study provided a basis for the identification of potential pathways and hub genes that might be involved in the PD-1 checkpoint blockade response microenvironment in influenza infection.

**Supplementary Information:**

The online version contains supplementary material available at 10.1186/s12863-022-01081-7.

## Background

Programmed cell death 1 (PD-1) is a negative checkpoint molecule that downregulates T cell activity after binding with its ligand, PD-1 ligand 1 (PD-L1). In chronic infections or tumors, PD-1 overexpression after lasting antigen-exposure will impair clearance of the pathogens or degenerate cells [1]. PD-1 blockade is already used as a successful therapy in multiple cancer treatments [2, 3]. The role of the PD-1/PD-L1 pathway in inhibiting immunity during chronic infections is well established [4]. Recently, its role in acute infections has aroused research attention [5].

Influenza virus, especially influenza A virus (IAV) infection, is a huge challenge to global public health, which, because of its high morbidity and mortality, and extremely high antigen mutation rate, has the possibility of causing epidemic outbreaks and even human-to-human transmission [6]. Severe infections often cause fatal pneumonia, which quickly leads to acute respiratory distress syndrome (ARDS) and multiple organ failure. Role of PD-1/PD-1 pathway in acute influenza infection has long been investigated [7, 8]. In recent years, studies have proven that acute influenza virus infection, especially severe infections, induce upregulated expression of the PD-1/PD-L1 pathway in an interferon receptor signaling-dependent manner, which leads to degranulation dysfunction and exhaustion of immune cells, especially CD8^+^ T cells [7].

The airway epithelium is the first barrier against influenza infection, which participates in host defense by producing cytokines and chemokines, and by regulating expression of surfactant proteins and adapter molecules. Experiments have confirmed that influenza virus infection can induce PD-1/PD-L1 signal overexpression and PD-1^+^ cell migration to the lung, which plays an important role in maintaining immune homeostasis [9, 10]. The spleen is the largest secondary immune organ and combines the innate and adaptive immune systems, which are important for antibacterial and antifungal immune reactivity. The spleen is a highly organized lymphoid compartment that removes blood-borne microorganisms and cellular debris. PD-1 and PD-L1 expression are high in the spleen [11] and upregulation of PD-1 expression correlated well with reduced gamma interferon (IFN-γ) and tumor necrosis factor (TNF) production after virus inoculation.

Using of immune checkpoint inhibitors in IAV infection is controversial, in addition to the enhanced viral immune response, it is not the whole picture, some researchers concern role of PD-1/PD-L1 pathway in developing autoimmune dilated cardiomyopathy with production of high-titer autoantibodies against cardiac troponin I after infection [12], some worried increasing the possibility of co-infection with other pathogens [13], the transcriptome reflects tissue activity at a given point in time, thus transcriptome expression studies provide an unbiased approach to investigate the PD-1 checkpoint blockade response during influenza infection.

## Methods

BALB/c mice (6 to 7 weeks old) were purchased from Joint Ventures SIPPER-BK Experimental Animal Co. (Shanghai, China). All animals were bred and maintained in specific pathogen-free conditions in accordance with the Care and Use of Laboratory Animals of Zhejiang Province and were approved by the local Ethics Committee. Six mice were divided into two groups: 1. The isotype control followed by A/PR8(H1N1) infection group (infection group, 50 μL 10^6^ median tissue culture infectious dose (TCID50) infective dose). 2. PD-1 antagonist followed with A/PR8(H1N1) infection group. The PD-1 antagonist comprised an antibody against PD-1 (clone RMP1–14; BioXCell, Lebanon, NH, USA), which was administered via tail vein injection in 200 μg doses on days 1, 4, and 7 before infection. An antibody against IgG2a (clone 2A3; BioXCell) was used as the isotype control. Mice were chemically restrained with 2,2,2-tribromoethanol (avertin) before intranasal challenge with 50 μL of 10^6^ TCID50 virus diluted in phosphate-buffered saline (PBS) [14, 15]. Mice were sacrificed 6 days after virus inoculation and their lungs and spleens were collected. Sixteen mice were grouped as above to observe the symptoms.

### Library preparation and sequencing for small RNAs

40–60 mg of lungs and spleens were homogenized by grinding in liquid nitrogen, and filled with TRIzol® reagent. After adding chloroform, the tubes were shaked vigorously for 15 s then incubated for 2–3 min. After centrifugation, the upper layer was transferred and added with isopropanol, and then centrifuged precipitate was washed with 75% alcohol. The RNA was dissolved in RNase-free water.

A total of 3 μg RNA per sample was used as input material, and sequencing libraries were generated using an NEB Next®Multiplex Small RNA Library Prep Set (NEB, Ipswich, MA, USA). Briefly, the NEB 3′ SR Adaptor was ligated to the 3′ end of microRNAs (miRNA), small interfering RNAs (siRNAs) and PIWI-interacting RNAs (piRNAs), then the SR RT Primer hybridized to the excess of 3′ SR Adaptor and transformed the single-stranded DNA adaptor into a double-stranded DNA molecule. PCR amplification was performed, and then the amplicons were purified. DNA fragments corresponding to 140 ~ 160 bp were recovered and dissolved. Finally, library quality was assessed on an Agilent Bioanalyzer 2100 system (Agilent, Santa Clara, CA, USA) using DNA High Sensitivity Chips.

The clustering of samples was performed on a cBot Cluster Generation System using TruSeq SR Cluster Kit v3-cBot-HS (Illumina, San Diego, CA, USA). After cluster generation, the library preparations were sequenced on an Illumina Hiseq 2500/2000 platform and 50 bp single-end reads were generated.

### Data analysis of small RNAs

As described before [16], mapped small RNA tags were used to looking for known miRNAs. miRBase20.0 was used as the reference, and the modified software mirdeep v2 and sRNA-tools-cli were used to obtain the potential miRNA and draw the secondary structures. The software miREvo v1.2 and mirdeep v2 were integrated to predict novel miRNAs. We followed the following priority rule: Known miRNA > rRNA > tRNA > snRNA > snoRNA > repeat > gene > NAT-siRNA > gene > novel miRNA > ta-siRNA to make every unique small RNA mapped to only one annotation. The known miRNAs used miFam.dat (http://www.mirbase.org/ftp.shtml) to look for families; novel miRNA precursors were submitted to Rfam (http://rfam.sanger.ac.uk/search/) to look for Rfam families. Predicting the target genes of the miRNAs was performed using miRanda v1.0b. Differential expression analysis was performed using the DESeq R package v1.8.3 with a *P*-value of 0.05 set as the threshold. The *P*-values was adjusted using the Benjamini & Hochberg method.

Gene Ontology (GO) enrichment analysis was used on the target gene candidates of the differentially expressed miRNAs. GOseq based Wallenius non-central hyper-geometric distribution which could adjust for gene length bias. GO enrichment analysis was implemented by the clusterProfiler R package v4.0 [17]. We used KOBAS v2.0 software to test the statistical enrichment of the target gene candidates in KEGG pathways [18, 19].

### Library preparation and sequencing for lncRNAs and mRNA

A total of 3 μg RNA per sample was used as input material to construct sequencing libraries, which were generated using the rRNA-depleted RNA by NEB Next® Ultra™ Directional RNA Library Prep Kit for Illumina®. The clustering of samples was performed on a cBot Cluster Generation System using TruSeq PE Cluster Kit v3-cBot-HS (Illumina), the libraries were sequenced on an Illumina Hiseq 4000 platform and 150 bp paired-end reads were generated.

### Data analysis of lncRNAs and mRNA

As described before [20], For lncRNA and mRNA, clean data were obtained by removing reads containing adapter, reads containing ploy-N and low quality reads from raw data. An index of the reference genome was built using bowtie2 v2.2.8 and paired-end clean reads were aligned to the reference genome using HISAT2 v2.0.4. The mapped reads of each sample were assembled using StringTie v1.3.1 in a reference-based approach.

All the transcripts were merged using Cuffmerge software. lncRNA and mRNAs were then identified from the assembled transcripts following four steps: (1) Removal of transcripts with uncertain chain directions; (2) Filtering the transcripts ≥200 bp and ≥ 2 exons; (3) Known mRNA and known lncRNA were identified by comparing the assembled transcripts with the reference genome GTF. (4) Filtering the transcripts with protein-coding capability using CNCI, Pfam and CPC2 database as Novel mRNA, filtering the transcripts without protein-coding capability using CNCI, Pfam and CPC2 database as Novel lncRNA.

Quantification of the transcripts was performed using StringTie software and Fragments Per Kilobase of transcript per Million mapped reads (FPKM) was obtained. EdgeR was used for differential expression analysis. All the transcripts were merged using Cuff merge software. Using hierarchical clustering method, lncRNA and mRNA are converted to log10 ^(FPKM + 1)^ values and clustered. Transcripts with *P* < 0.05 were assigned as differentially expressed. GO enrichment analysis and KEGG pathway enrichment analysis were performed as above.

### Quantitative reverse transcription RT-PCR analysis (qRT-PCR)

Approximately 10 differentially expressed transcripts identified among the two groups of samples were verified by qRT-PCR using three biological replicates. Maize tubulin was used as an internal reference gene. The relative expression levels of each gene were calculated using the 2-∆∆CT method.

## Results

### Evaluation of weight changes in the experimental groups

**Fig. 1:**
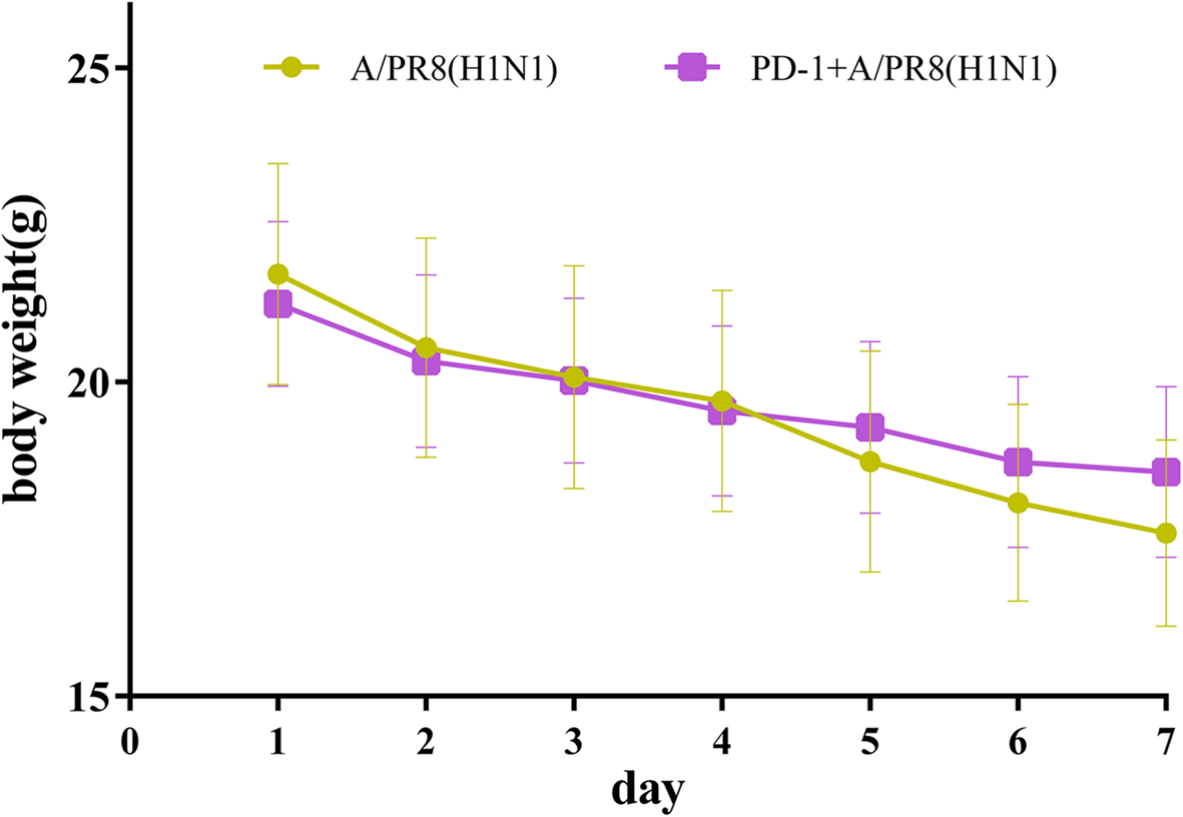
Weight changes of the mice Weight changes of the mice (each group had 8 mice) after IAV infection, wildtype IAV was challenged on day 1

We observed lethargy, ruffled fur, and loss of appetite, but no death in any IAV-challenged mice, the degree of weight loss of these two groups with 8 mice per group were shown in Fig. 1 (*P* = 0.006). The viral load and pathological damage of lung tissue 6 day post infection are depicted in Supplementary Figs. 1 and 2, respectively. Anti-PD-1 antibody treatment significantly alleviated the severity of necrosis and inflammation based on the gross and microscopic lesions and decreased the viral titers in the lungs.

### Differential expression analysis

**Fig. 2:**
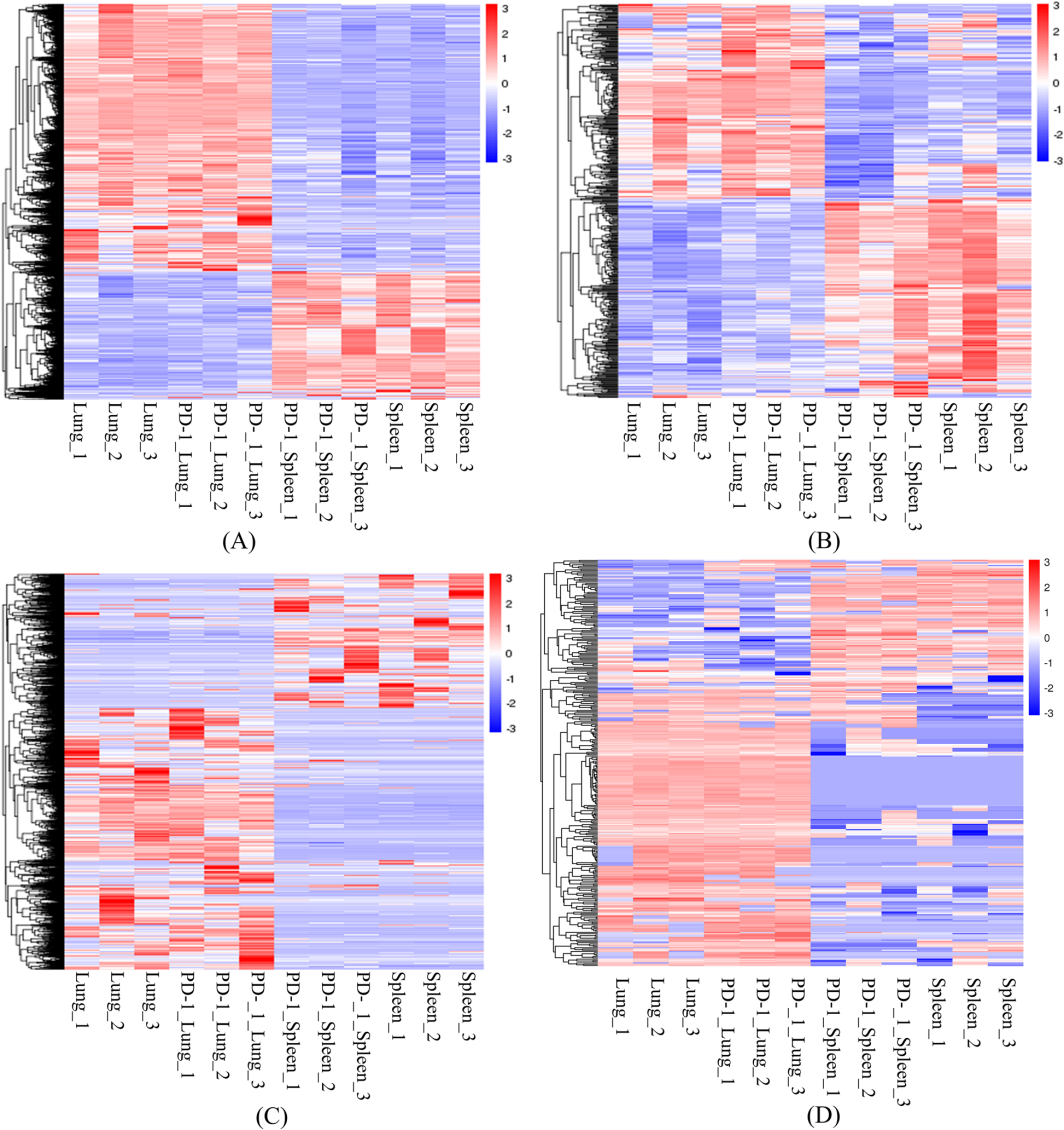
Heatmaps of differentially expressed transcripts. Heatmaps of differentially expressed mRNAs (**A**), differentially expressed miRNAs (**B**), differentially expressed lncRNAs (**C**), and differentially expressed circRNAs (**D**) of lungs and spleens of the following groups: PD-1 antagonist treatment followed by A/PR8(H1N1) infection group vs. isotype control followed by A/PR8(H1N1) infection group. Red indicates upregulation, and blue indicates downregulation

In the differential expression analysis of lungs (Fig. 2), 85 differentially expressed mRNAs (dif-mRNAs) were obtained of which 76 were upregulated and 9 were downregulated, functional genes in PD-1 antagonist treatment followed by A/PR8(H1N1) infection group included Nppa, Myl7, Car3, Itprip, CAMP (log2FoldChange of 11.5, 2.5, 5.7, 2.5 and 3.4 respectively) et al.; 36 differentially expressed miRNAs (dif-miRNAs) were identified, of which 19 were upregulated and 17 were downregulated; 90 differentially expressed lncRNAs (dif-lncRNAs) were obtained, including 70 upregulated and 20 downregulated; and 22 differentially expressed circRNAs (dif-circRNAs) were found, of which 13 were upregulated and 9 were downregulated.

In the spleen data, 45 dif-mRNAs were obtained, of which 18 were upregulated and 27 were downregulated, functional genes in PD-1 antagonist treatment followed by A/PR8(H1N1) infection group included CAMP, Ltf, Ly6g, Bola1 (log2FoldChange of 2.1, 2, 2.6, and 2.5 respectively) et al.; 36 dif-miRNAs were identified, of which 19 were upregulated and 17 were downregulated; 57 dif-lncRNAs were obtained, including 22 upregulated and 35 downregulated; and 24 dif-circRNAs were found, of which 18 were upregulated and 6 were downregulated.

### Functional enrichment analysis of dif-mRNAs and dif-miRNAs in lungs and spleens

**Fig. 3:**
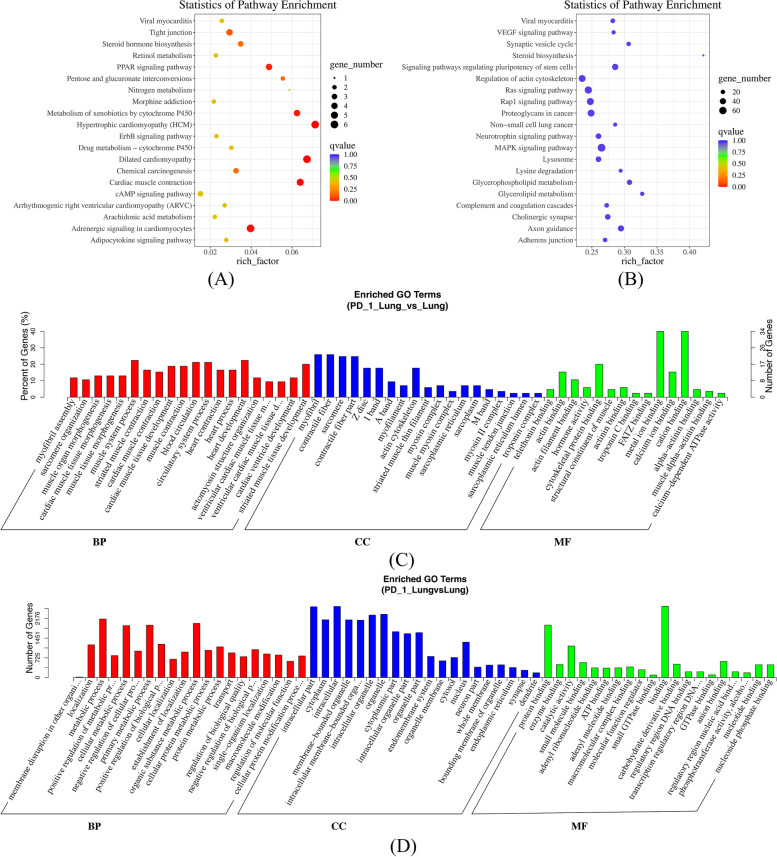
Gene Ontology (GO) and KEGG pathway analysis of dif-mRNAs and dif-miRNAs in the lungs. The top 20 pathways and GO terms (BP (Biological Process), CC (cellular component), and MF (molecular function)) enriched by 85 dif-mRNAs and 36 dif-miRNAs of lungs of the following groups: PD-1 antagonist treatment followed by A/PR8(H1N1) infection group vs. isotype control followed by A/PR8(H1N1) infection group. **A** Top 20 pathways enriched by dif-mRNAs. **B** Top 20 pathways enriched by dif-miRNAs. **C** Top 20 GO terms enriched by dif-mRNAs. **D** Top 20 GO terms enriched by dif-miRNAs

**Fig. 4:**
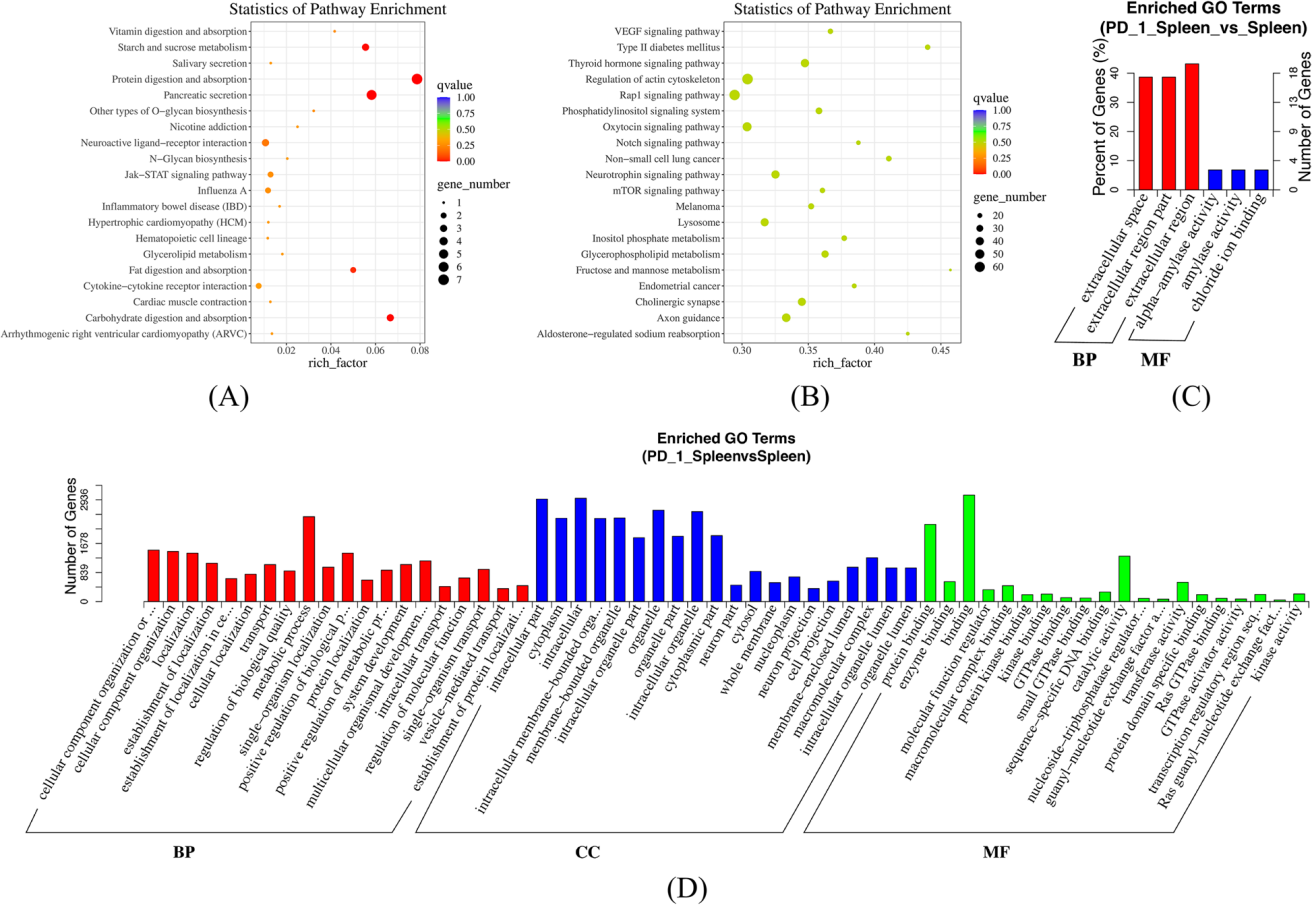
GO and KEGG pathway analysis of dif-mRNAs and dif-miRNAs in spleens

KEGG and GO analyses were used to investigate the functional associations of gene expression changes. Targeted genes of dif-mRNA and dif-miRNAs of lungs and spleens of the two groups: PD-1 antagonist followed with A/PR8(H1N1) infection group vs. Isotype control followed with A/PR8(H1N1) infection were predicted (Figs. 3 and 4). The gene lists used in the dif-mRNAs analysis contained 18,455 and 17,818 genes for lungs and spleens, respectively. 1290 and 1290 genes were analyzed for lungs and spleens for dif-miRNAs. For GO, biological process, cellular component, and molecular function were selected as the annotation categories for clustering. Once the tool identified enriched ontologies for a particular gene list, it clusters those that have a statistically significant overlap in terms of their constituent genes. *P*-value was set < 0.05, the dif-mRNAs were enriched in 11 pathways in lungs and 6 pathways in spleens. Dif-miRNAs were enriched in 11 pathways in lungs and 26 pathways in spleens. There was little degree of overlap of dif-mRNAs and dif-miRNAs in lungs between the most enriched clusters. The most enriched clusters of dif-mRNAs of lungs were related to muscle and heart biological behavior. More than 85% of the dif-miRNAs enriched clusters in lungs and spleens overlapped with each other, including localization, metabolic process, positive regulation of metabolic process, and regulation of molecular function in the biological process category; intracellular part, cytoplasm, intracellular, and membrane-bounded organelle in the cellular component category; and protein binding, enzyme binding, and molecular function regulator in the molecular function category.

Functional Enrichment analysis of mRNAs and miRNAs in lungs and spleens obtained from IAV infection mice treated with anti-PD-1 antibody clearly highlighted myocardial damage related to viral infection, mitogen-associated protein kinase (MAPK) signaling pathways, RAP1 (Ras-related protein 1) signaling pathway, and Axon guidance.

The top 20 pathways and GO terms (BP (Biological Process), CC (cellular component), and MF (molecular function)) enriched by 45 dif-mRNAs and 36 dif-miRNAs of spleens of the following groups: PD-1 antagonist treatment followed by A/PR8(H1N1) infection group vs. isotype control followed by A/PR8(H1N1) infection group. (A) Top 20 pathways enriched by dif-mRNAs (B) Top 20 pathways enriched by dif-miRNAs (C) Top 20 GO terms enriched by dif-mRNAs (D) Top 20 GO terms enriched by dif-miRNAs.

### Enrichment analysis of lncRNA and circRNA-related target genes

**Fig. 5:**
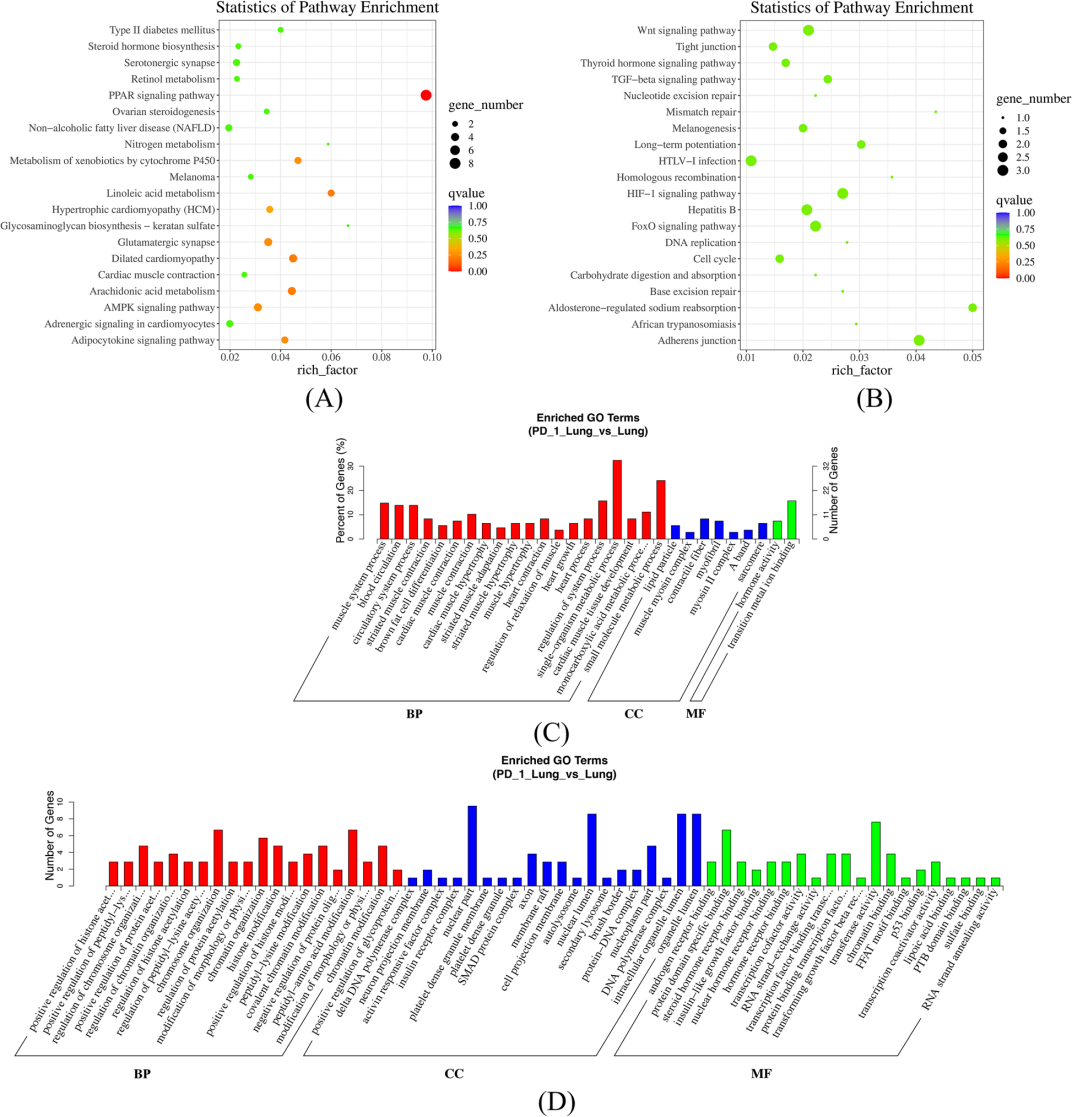
Analysis of GO and KEGG pathways of dif- lncRNAs and dif-circRNAs of the lungs

**Fig. 6:**
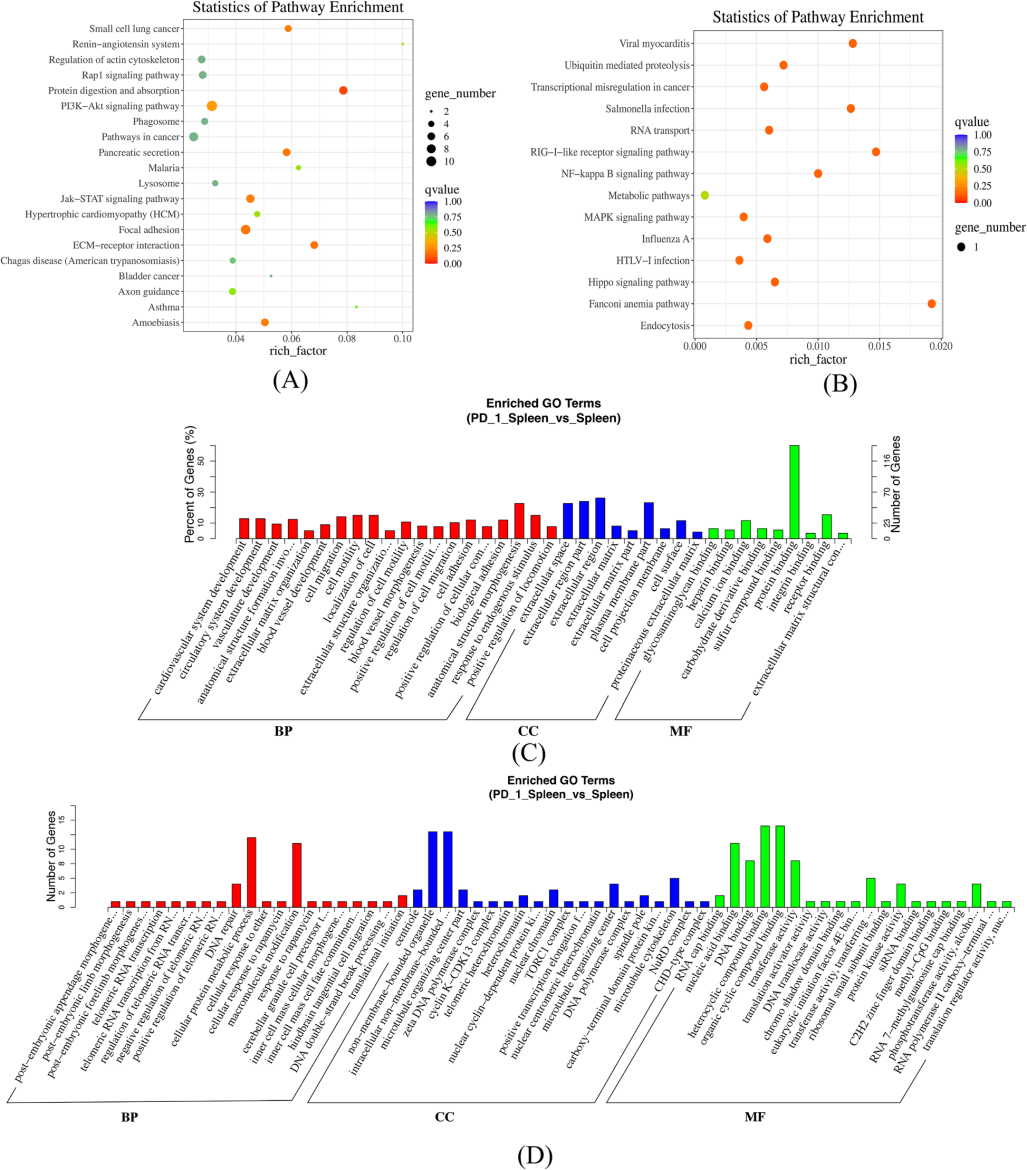
Analysis of GO and KEGG Pathway of dif- lncRNAs and dif-circRNAs of spleens

KEGG and GO analysis was performed for dif-lncRNA and dif-circRNA-related target genes (Figs. 5 and 6). *P*-value was set < 0.05, the dif- lncRNA target genes were enriched in 9 pathways in lungs and 12 pathways in spleens. The dif- circRNA target genes were enriched in 7 pathways in lungs and 4 pathways in spleens. There was a little degree of overlap of lncRNAs and circRNAs in lungs and spleens between the most enriched clusters except for Hypertrophic cardiomyopathy, MAPK signaling pathway, and the AMP-activated protein kinase (AMPK) signaling pathway.

Top 20 pathways and GO terms (BP (Biological Process), CC (cellular component), and MF (molecular function)) enriched by 90 dif- lncRNAs and 22 dif-circRNAs of the lungs (A) Top 20 pathways enriched by dif-lncRNAs. (B) Top 20 pathways enriched by dif-circRNAs (C) Top 20 GO terms enriched by dif-lncRNAs (D) Top 20 GO terms enriched by dif-circRNAs.

Top 20 pathways and GO terms (BP (Biological Process), CC (cellular component), and MF (molecular function)) enriched by 57 dif- lncRNAs and 24 dif-circRNAs of spleens (A) Top 20 pathways enriched by dif-lncRNAs. (B) Top 20 pathways enriched by dif-circRNAs. (C) Top 20 GO terms enriched by dif-lncRNAs (D) Top 20 GO terms enriched by dif-circRNAs.

### Competing endogenous RNA network construction

According to the dif-lncRNA–dif-miRNA pairs and dif-miRNA–dif-mRNA pairs, differentially expressed lncRNAs and mRNAs regulated by the same miRNA were screened. In total, 77 lncRNA-miRNA-mRNA interactions in lungs were finally obtained (Supplementary Fig. 1), including 35 upregulated lncRNAs and 9 downregulated lncRNAs, 5 upregulated and 5 downregulated mRNAs, and 2 upregulated and 5 downregulated miRNAs. In spleens, 131 lncRNA-miRNA-mRNA interactions were finally obtained (Supplementary Fig. 2), including 29 upregulated lncRNAs and 26 downregulated lncRNAs, 17 upregulated and 8 downregulated mRNAs, and 5 upregulated and 4 downregulated miRNAs.

Two interaction relationships of circRNA-miRNA-mRNA in lungs were obtained (Supplementary Fig. 3), comprising 2 upregulated circRNAs, 2 upregulated mRNAs, and 1 downregulated miRNA. In spleens, 32 interaction relationships of circRNA-miRNA-mRNA were obtained (Supplementary Fig. 4) including 6 upregulated circRNAs and 1 downregulated circRNA, 16 upregulated mRNAs and 2 downregulated mRNAs, 2 upregulated miRNAs and 4 downregulated miRNAs.

**Fig. 7:**
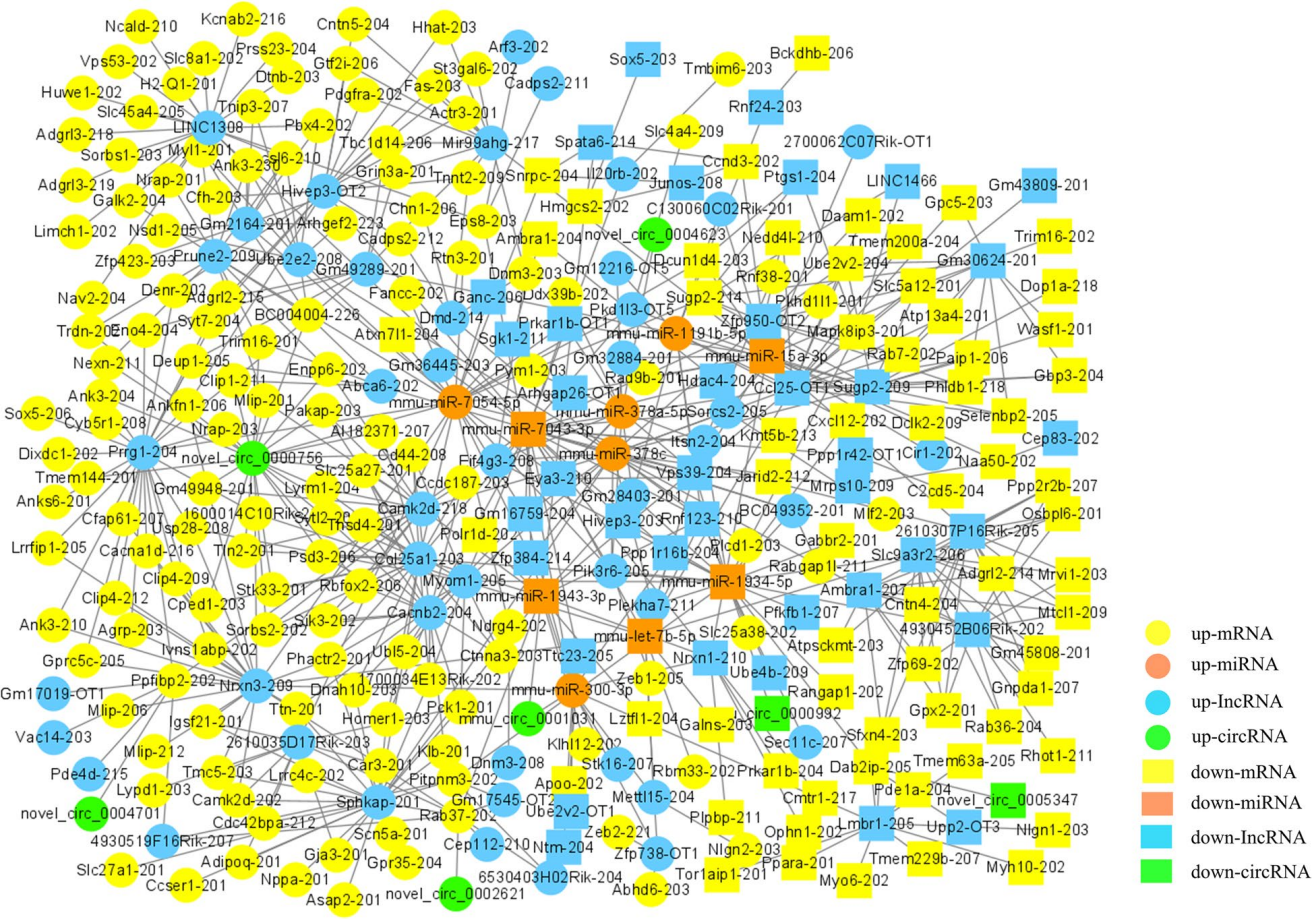
The Competing Endogenous RNA (ceRNA) network of the lungs. Circles represent upregulation and rectangles represent downregulation. mRNAs, miRNAs, lncRNAs, and circRNAs in the network are presented in yellow, orange, blue, and green, respectively

**Fig. 8:**
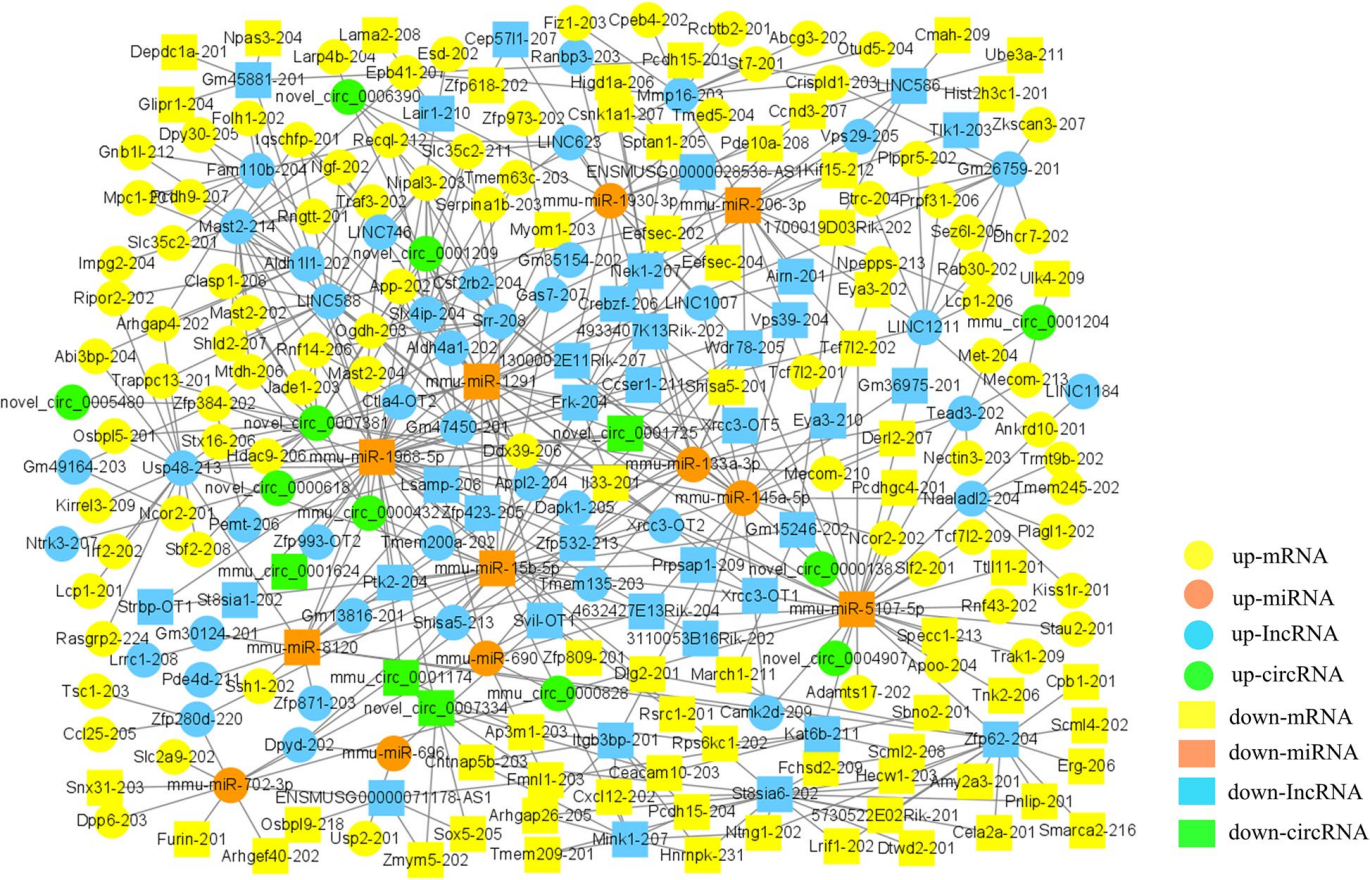
The Competing Endogenous RNA (ceRNA) network of the spleens. Circles represent upregulation and rectangles represent downregulation. mRNAs, miRNAs, lncRNAs, and circRNAs in the network are presented in yellow, orange, blue, and green, respectively

Further, differentially expressed circRNAs, lncRNAs, and mRNAs that were regulated by the same miRNA were further screened based on the lncRNA-miRNA-mRNA and circRNA-miRNA-mRNA analysis. Finally, 595 interaction pairs were obtained in lungs (Fig. 7), comprising 135 upregulated and 63 downregulated mRNAs, 5 upregulated and 5 downregulated miRNAs, 5 upregulated and 2 downregulated circRNAs, and 46 upregulated and 38 downregulated lncRNAs. There were 462 interaction pairs in spleens (Fig. 8), comprising 85 upregulated and 64 downregulated mRNAs, 6 upregulated and 6 downregulated miRNAs, 42 upregulated and 36 downregulated circRNAs, and 10 upregulated and 4 downregulated lncRNAs. Downregulated mmu-miR-7043-3p and Vps39–204 were significantly enriched in the ceRNA network.

### Validation by qRT-PCR

**Fig. 9:**
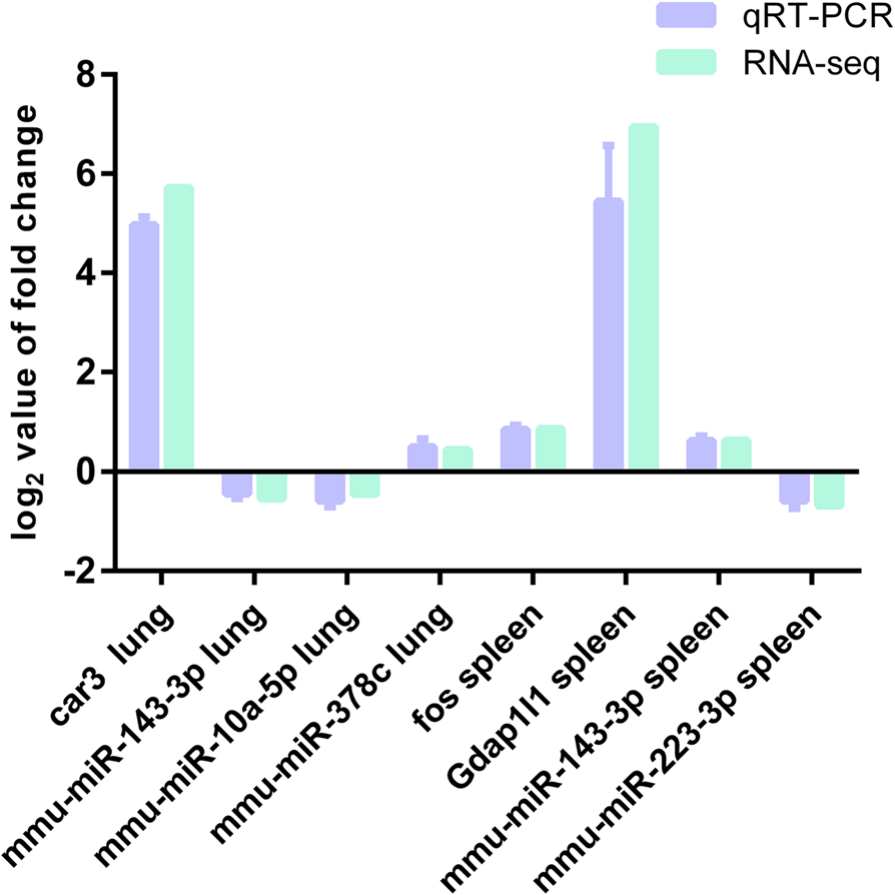
Quantitative RT-PCR validation of differentially expressed transcripts. Error bars indicate standard error of three replicates

The expression levels determined by qRT-PCR were in agreement with the changes in transcript abundance determined by RNA-seq analysis, which suggested that our transcriptome profiling data were highly reliable (Fig. 9).

## Discussion

The PD-1/PD-L1 signaling pathway has important regulatory roles in antiviral responses, and PD-1/PD-L1 upregulation is induced by persistent viruses, including human immunodeficiency virus (HIV) [21, 22], hepatitis C virus (HCV) [23], and hepatitis B virus (HBV) [24], which impairs T cell responses and is unfavorable for virus clearance. Upregulated PD-1/PD-L1 expression induced by influenza A virus infection is an important component of the immunosuppressive microenvironment, and blocking this signaling pathway may reduce tissue damage, lower virus titers in the lung, and alleviate symptoms of infection to promote recovery [7, 9, 25]. Other potential molecular biology changes is important, however, the molecular mechanism of the PD-1 checkpoint in acute infection are still not well understood and are thus worthy of in-depth investigation.

In this study, by applying whole-transcriptome sequencing, we identified 84 dif-mRNAs, 36 dif-miRNAs, 90 dif-lncRNAs, and 22 dif-circRNAs in PD-1 antagonist treated A/PR8(H1N1) influenza infected lungs compared with those in the controls (IgG2a isotype control treated before infection). In the comparison between the spleen samples from the above two groups, 45 dif-mRNAs, 36 dif-miRNAs, 57 dif-lncRNAs, and 24 dif-circRNAs were identified. Direct functional enrichment analysis on the dif-mRNAs and dif-miRNAs showed that these genes were mainly involved in myocardial damage related to viral infection, MAPK signaling pathways, the RAP1 signaling pathway, and Axon guidance.

Functional Enrichment analysis of mRNA and miRNA in lung and spleen clearly highlighted myocardial damage related to viral infection. Influenza virus is an etiological agent of myocarditis, and the relationship between acute respiratory virus infection, especially influenza, and associated viral myocardial damage is greatly underestimated. Many studies have reported that influenza virus infection, especially severe infection, causes fatal myocarditis in humans and experimental animals. Acute cardiovascular events even death triggered by influenza was first noted as early as the 1930s. Several studies have confirmed that acute respiratory infections or influenza-like illnesses were closely related to subsequent acute cardiovascular events [26, 27], viruses might replicate in the heart of at least 10% of patients with infection, and pathological injuries include focal infiltration with inflammatory cells in the interstitial and pericardium areas, myocardial edema, and cardiac necrosis. The basic treatment is hemodynamic and ventilatory support; however, the use of immunosuppressive or antiviral therapy for fulminant myocarditis of viral etiology is controversial [28]. Our sequencing result suggested that PD-1 antagonist may aggravate virus-induced cardiomyocyte damage, however, this conclusion needs to be further confirmed in a larger scale animal experiment.

The MAPK signaling pathway plays an important role in regulating cell proliferation, differentiation, invasion, metastasis, and death through phosphorylation activation. The relationship between MAPK signaling pathways and anti-PD-1 antibody in infectious disease has been discussed elsewhere, especially in chronic infection. MAPK activation is an important initiating event in the upregulation of PD-1 in HIV-1-infected cells, and inhibition of this signaling pathway can reduce infection [29]. The HA protein of influenza A virus is conserved among strains and subsets, and axon guidance molecules were proven to have a large pentapeptide overlap, thus immune cross-reactivity between influenza HA and axon guidance molecules is possible [30–32]. PD-1 signaling inhibits Rap guanine nucleotide exchange factor 1 (RAPGEF1 also known as C3G) phosphorylation by utilizing SHP-1/2 (also known as protein tyrosine phosphatase non-receptor type 6 and type 11), and reduced levels of phosphorylated C3G result in reduced RAP1 activation and adhesion to intercellular adhesion molecule 1 (ICAM-1) to inhibit T-cell adhesion. Several studies suggested that sepsis-induced upregulation of PD-1 has an impact on the motility and migratory capacity of T lymphocytes by regulating classical inhibitory motif recruitment, activation of the phosphatases SHP-1/2, and signaling through RAP1 [33].

Additionally, we identified the significant role of downregulated mmu-miR-7043-3p and Vps39–204 in the ceRNA network. Decreased expression of mmu-miR-7043-3p was proven to be one of remarkable miRNA signatures of myocardial reductive stress, which is associated with cardiac hypertrophy [34]. Future mechanistic studies are needed to determine the role of miR-7043-3p in PD-1/PD-L1 pathway-associated viral damage in influenza infection. VPS39 is a member of the vacuolar tethering complex that promotes late endosome formation, and evidence has shown that silencing VPS39 can increase the proliferation of aged human T cells and memory responses of lysosome-defective T cells in a mouse viral infection model [35], and thus might play important roles in antiviral immunity.

## Conclusions

In conclusion, this study explored the molecular mechanism of the PD-1 checkpoint blockade response microenvironment during influenza infection. Upregulated PD-1/PD-L1 expression-induced by IAV infection is an important component of the immunosuppressive microenvironment, and blocking this signaling pathway will regulate the following signal pathways: Myocardial damage related to viral infection, MAPK signaling pathways, Rap1 signaling pathway, and Axon guidance. Downregulated mmu-miR-7043-3p and Vps39–204 were most significantly enriched by PD-1 blockade. However, this study was limited by a small sample size and limited time points to provide a comprehensive overview of the PD-1 checkpoint response microenvironment. Further in vivo validation using a larger scale animal experiment and dynamic functional characterization are needed to delineate the exact mechanistic details.

## Supplementary Information

**Additional file 1: Supplementary Figure 1.** Viral titers of the lungs 6 day post infection. Viral titers of the lungs 6 days after wildtype IAV challenge (each group had 8 mice). Viral replication in the lungs of IAV-challenged BALB/c mice was determined using the TCID50 method in MDCK cells. Viral titers were expressed as the means ± SE of the log10 TCID50 per gram of tissue. ***P < 0.0001.

**Additional file 2: Supplementary Figure 2.** The lung histopathology of mice after virus infection. The lung histopathology of mice treated with or without a PD-1 antagonist and subsequently intranasally inoculated with 10^6^ TCID50 A/PR8 at 6 day post infection. Multiple 4-μm-thick sections were stained with H&E. Original magnification: 200×. Three independent pathologists calculated the inflammatory pathology score of lung tissues according to the standard of Underwood, which including perivascular and peribronchiolar eosinophilia, edema and epithelial damage. Comparing A/PR8(H1N1) infection group with PD-1 antagonists treated A/PR8(H1N1) infection group, pathology scores were 10 ± 0.6 to 7.8 ± 0.4.

**Additional file 3: Supplementary Figure 3.** The lncRNA-miRNA-mRNA network of the lungs. Circles represent upregulation and rectangles represent downregulation. mRNAs, miRNAs, and lncRNAs in the network are presented in yellow, orange, and green, respectively.

**Additional file 4: Supplementary Figure 4.** The lncRNA-miRNA-mRNA network of the spleens. Circles represent upregulation and rectangles represent downregulation. mRNAs, miRNAs, and lncRNAs in the network are presented in yellow, orange, and green, respectively.

**Additional file 5: Supplementary Figure 5.** The circRNA-miRNA-mRNA network of the lungs. Circles represent upregulation and rectangles represent downregulation. mRNAs, miRNAs, and circRNAs in the network are presented in yellow, orange, and green, respectively.

**Additional file 6: Supplementary Figure 6.** The circRNA-miRNA-mRNA network of the spleens. Circles represent upregulation and rectangles represent downregulation. mRNAs, miRNAs, and circRNAs in the network are presented in yellow, orange, and green, respectively.

## Data Availability

The datasets generated and analyzed during the current study are available in the Gene Expression Omnibus (GEO) repository: https://www.ncbi.nlm.nih.gov/geo/query/acc.cgi?acc=GSE192916(accession number: GSE192916).
