# Supplementary figures and images for: Bioinformatic analysis of PD-1 checkpoint blockade response in influenza infection
Source: BMC Genom Data. 2022 Aug 13;23:65. doi: 10.1186/s12863-022-01081-7 (PMC9374577; doi:10.1186/s12863-022-01081-7)

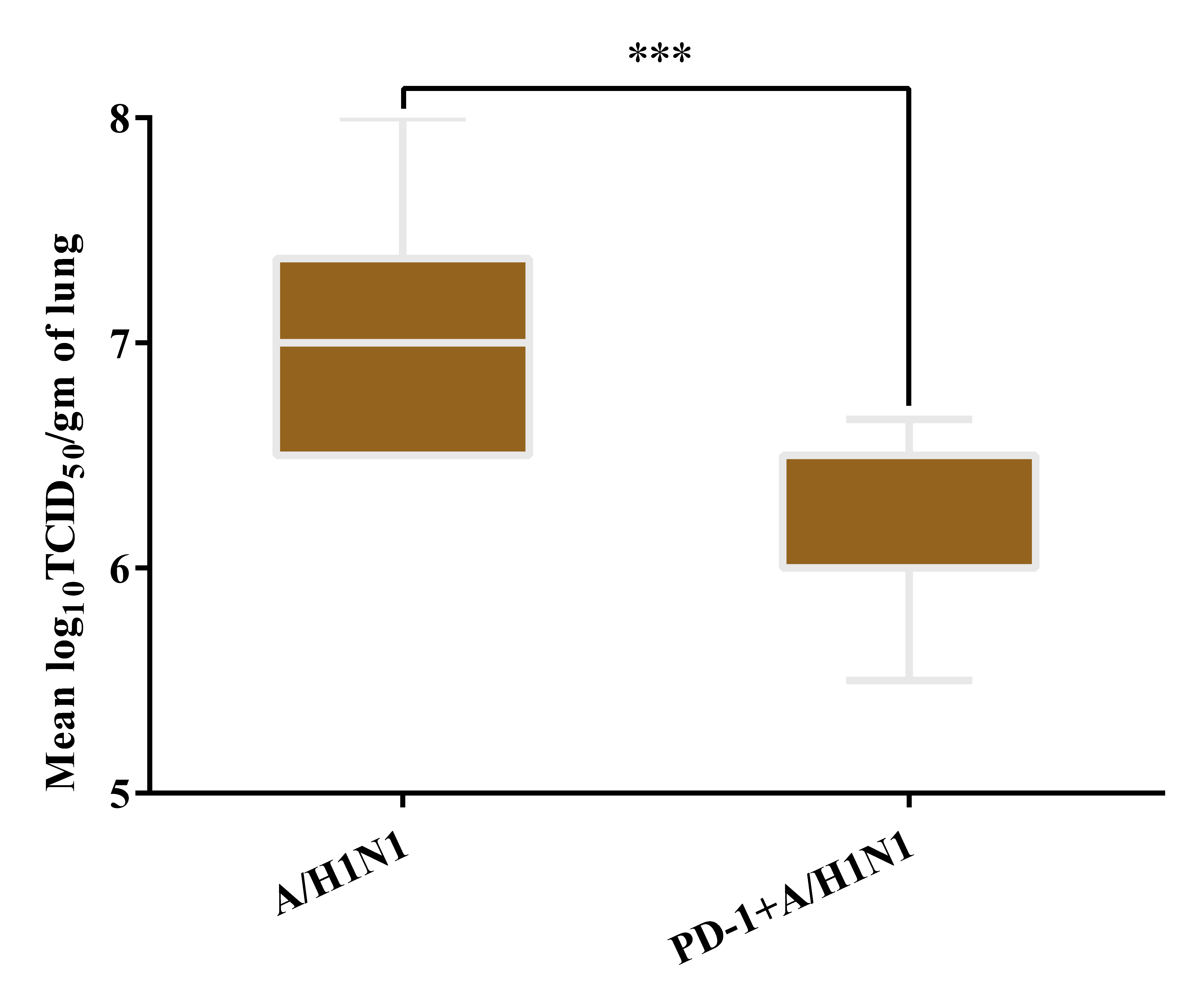

Supplement: Supplementary file 1 — Additional file 1: Supplementary Figure 1. Viral titers of the lungs 6 day post infection. Viral titers of the lungs 6 days after wildtype IAV challenge (each group had 8 mice). Viral replication in the lungs of IAV-challenged BALB/c mice was determined using the TCID50 method in MDCK cells. Viral titers were expressed as the means ± SE of the log10 TCID50 per gram of tissue. ***P < 0.0001. [file 12863_2022_1081_MOESM1_ESM.png]

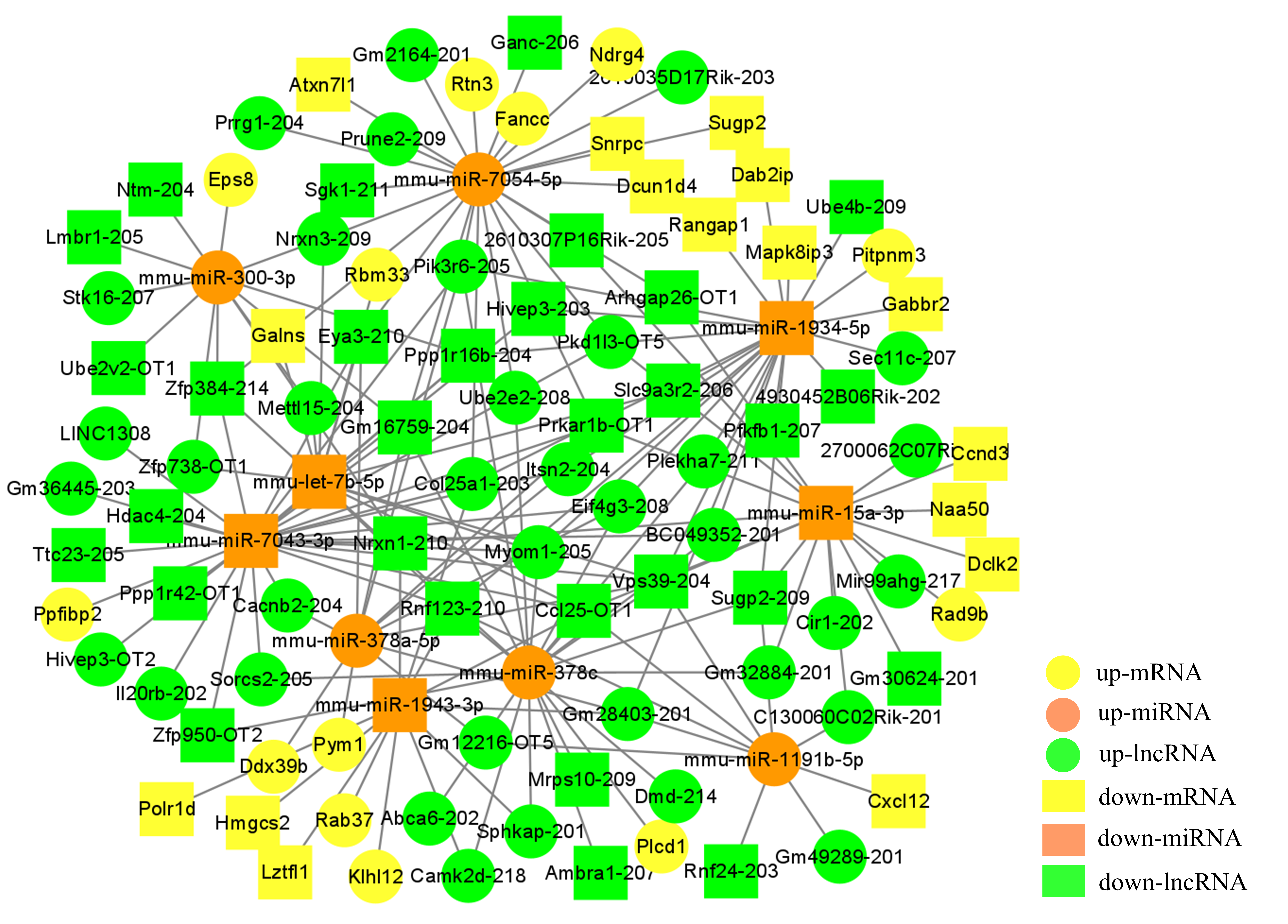

Supplement: Supplementary file 3 — Additional file 3: Supplementary Figure 3. The lncRNA-miRNA-mRNA network of the lungs. Circles represent upregulation and rectangles represent downregulation. mRNAs, miRNAs, and lncRNAs in the network are presented in yellow, orange, and green, respectively. [file 12863_2022_1081_MOESM3_ESM.png]

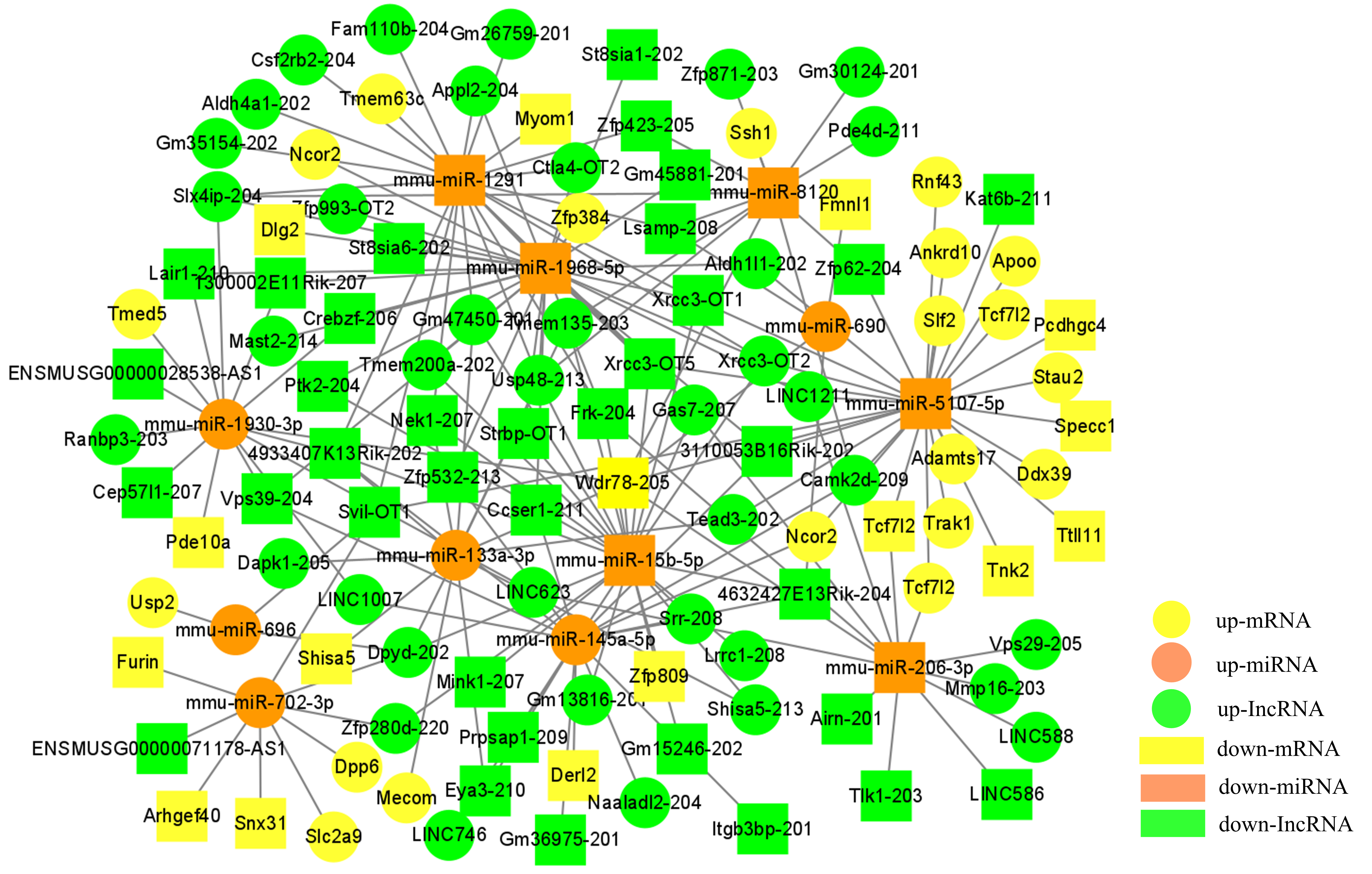

Supplement: Supplementary file 4 — Additional file 4: Supplementary Figure 4. The lncRNA-miRNA-mRNA network of the spleens. Circles represent upregulation and rectangles represent downregulation. mRNAs, miRNAs, and lncRNAs in the network are presented in yellow, orange, and green, respectively. [file 12863_2022_1081_MOESM4_ESM.png]

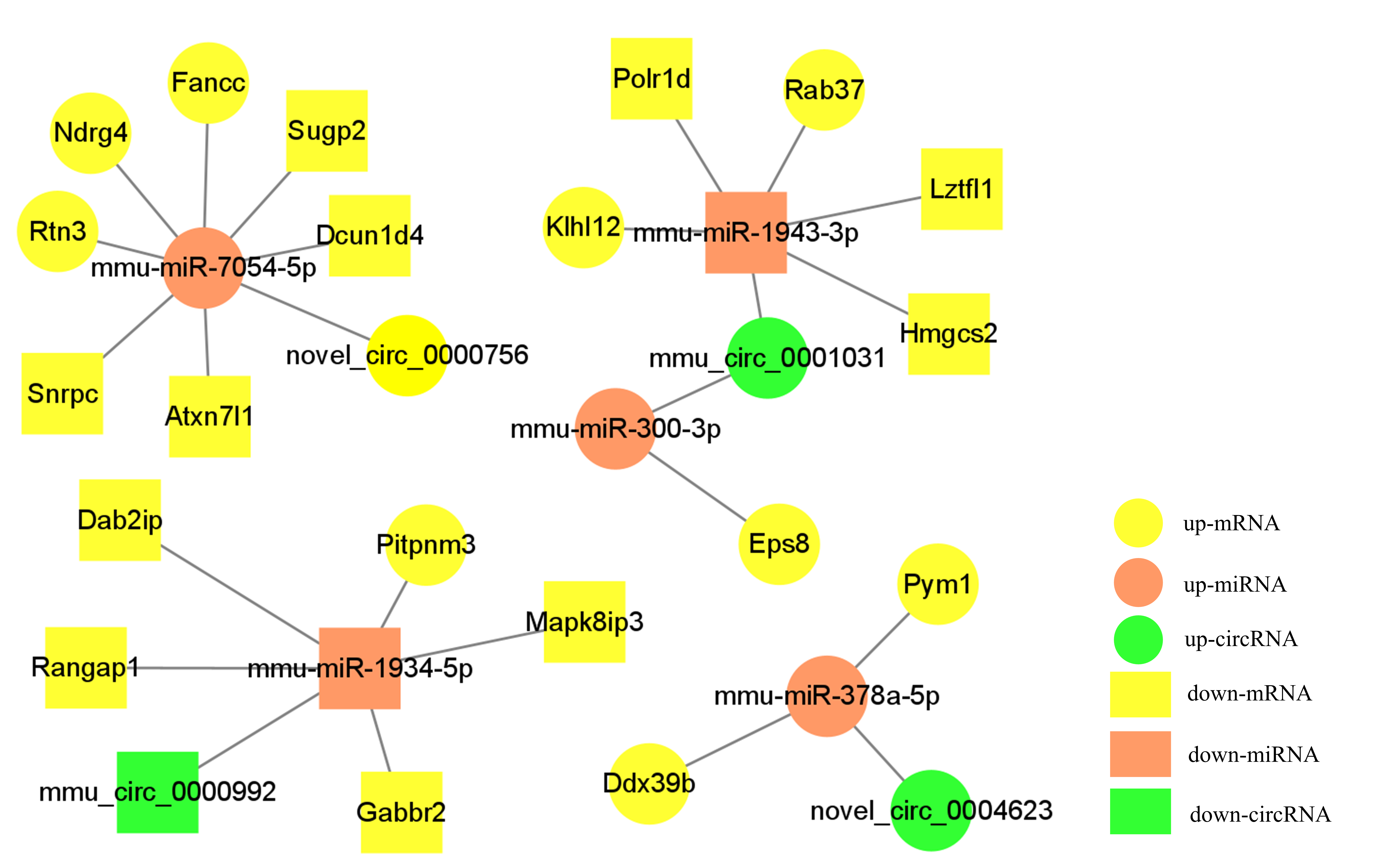

Supplement: Supplementary file 5 — Additional file 5: Supplementary Figure 5. The circRNA-miRNA-mRNA network of the lungs. Circles represent upregulation and rectangles represent downregulation. mRNAs, miRNAs, and circRNAs in the network are presented in yellow, orange, and green, respectively. [file 12863_2022_1081_MOESM5_ESM.png]

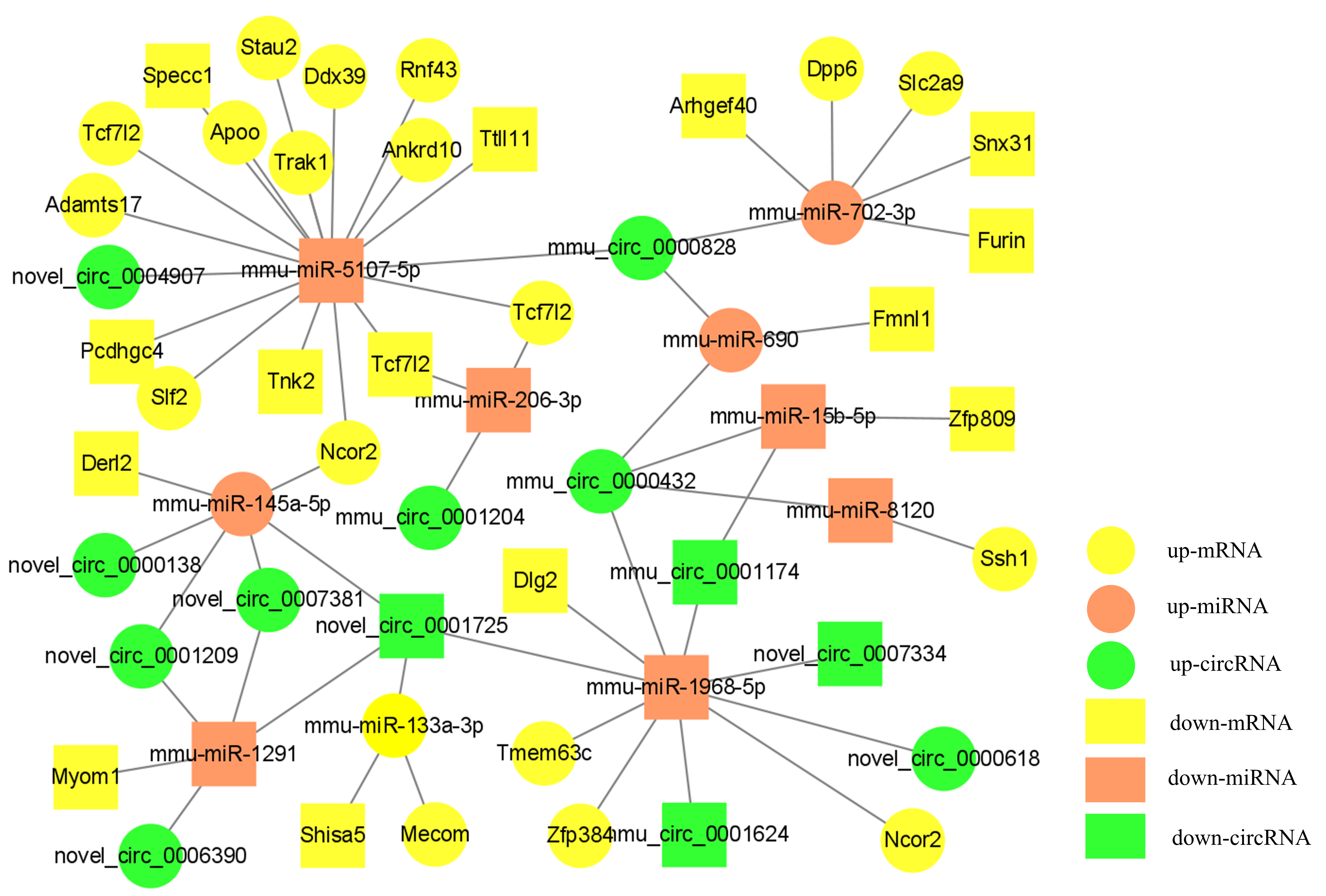

Supplement: Supplementary file 6 — Additional file 6: Supplementary Figure 6. The circRNA-miRNA-mRNA network of the spleens. Circles represent upregulation and rectangles represent downregulation. mRNAs, miRNAs, and circRNAs in the network are presented in yellow, orange, and green, respectively. [file 12863_2022_1081_MOESM6_ESM.png]
